# Cardiac pericytes function as key vasoactive cells to regulate homeostasis and disease

**DOI:** 10.1002/2211-5463.13021

**Published:** 2020-12-14

**Authors:** Linda L. Lee, Aarif Y. Khakoo, Vishnu Chintalgattu

**Affiliations:** ^1^ Department of Cardiometabolic Disorders Amgen Research and Discovery Amgen Inc. South San Francisco CA USA; ^2^ Department of Drug Development Calico Labs South San Francisco CA USA

**Keywords:** cardiac pericyte, heart, pericytes, perivascular cells, vascular biology

## Abstract

Pericytes (PCs)—mural cells that envelop endothelial cells (ECs) of microvessels—regulate tissue‐specific vasculature development as well as maturation and maintenance of endothelial barrier integrity. However, little is known about their tissue‐specific function in the heart. Specifically, the mechanism by which cardiac PCs constrict coronary capillaries remains undetermined. To gain insights into the function of cardiac PCs at the cellular level, we isolated NG2^+^ PDGFRβ^+^ CD146^+^ CD34^−^ CD31^−^ CD45^−^ PCs for detailed characterization. Functionally, we provide evidence that these PCs increased transepithelial electrical resistance and decreased endothelial permeability. We show for the first time that this population of PCs express contractile proteins, are stimulated by adrenergic signaling, and demonstrate stereotypical contraction and relaxation. Furthermore, we also studied for the first time, the PCs in *in vitro* models of disease. PCs in hypoxia activated the hypoxia‐inducible factor 1 alpha pathway, increased secretion of angiogenic factors, and caused cellular apoptosis. Supraphysiological levels of low‐density lipoprotein decreased PC proliferation and induced lipid droplet accumulation. Elevated glucose levels triggered a proinflammatory response. Taken together, our study characterizes cardiac PCs under *in vitro* disease conditions and supports the hypothesis that cardiac PCs are key vasoactive cells that can regulate blood flow in the heart.

## Graphical abstract

Pericytes (PCs) in general are known to help maintain vessel integrity; however, their specific function in the heart is not well defined. Our *in vitro* study details NG2^+^ PDGFRβ^+^ CD146^+^ CD34^−^ CD31^−^ CD45^−^ cardiac PC phenotypic and functional characteristics, and their role in vasoregulation in physiological and disease prone environments. Cardiac PCs can potentially regulate blood flow in the heart.

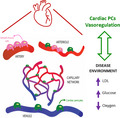

**Abbreviations**
ADFP: adipose differentiation‐related protein
CCL2: C‐C motif chemokine ligand 2
DMEM: Dulbecco’s modified Eagle media
EC: endothelial cell
EGM‐2: endothelial growth medium 2
FACS: fluorescence‐activated cell sorting
HIF‐1α: hypoxia‐inducible factor 1 alpha
LDL: low‐density lipoprotein
MI: myocardial infarction
NG2: neural glial 2
P/S: penicillin/streptomycin
PC: pericyte
PDGFbb: platelet‐derived growth factor bb
PDGFRβ: platelet‐derived growth factor receptor beta
PE: phenylephrine
SMC: smooth muscle cell
TEER: transepithelial electrical resistance
TNF‐α: tumor necrosis factor alpha
VEGF‐A: vascular endothelial growth factor‐A
α‐SMA: alpha smooth muscle actin

Pericytes (PCs) are mural cells that constitute part of the vascular system and are found on the abluminal side of capillaries and microvessels [1, 2]. Phenotypically, PCs have a round flat body with extensions that wrap around endothelial cells (ECs) to form tight junctions. These cells are identified by extensively validated markers such as neural glial 2 (NG2) [3, 4], and platelet‐derived growth factor receptor beta (PDGFRβ) [3, 5, 6] as well as alpha smooth muscle actin (α‐SMA) [7], desmin [8, 9, 10], calponin [3], and vimentin [11, 12]. PCs take part in angiogenesis, stabilize, and mature newly sprouted vessels, and help maintain vascular integrity [1, 5, 13, 14]. Dysregulation and/or loss of PCs can lead to decreased endothelial barrier integrity and vascular dysfunction [5, 15, 16, 17, 18, 19]. Moreover, PC dysregulation has been implicated in the progression of multiple diseases such as Alzheimer's disease, fibrosis, diabetic neuropathy, osteogenesis, tumor angiogenesis, and atherosclerosis [15, 20, 21, 22, 23, 24, 25, 26, 27]. PC biology is well defined in the brain, the retina, and skeletal muscle; however, little is known about their role in the heart and how they contribute to cardiac homeostasis and hemodynamics under both normal physiological and pathophysiological conditions.

Cardiac PC biology is an emerging topic in the field, and few laboratories have published on cardiac PCs [3, 11, 28]. Functionally, previous studies have shown that cardiac PCs form and support networks with ECs in a coculture system *in vitro*. Like in other organs, cardiac PCs play an essential role in maintaining vessel homeostasis and integrity. Due to their anatomical location, PCs have been hypothesized to control capillary blood flow and pressure. These cells behave as pre‐ and postcapillary sphincters that regulate perfusion in the smallest unit of the vascular tree. The ability of PCs to contract and relax has been studied on retinal [29, 30], renal [31, 32, 33, 34], and brain [35, 36, 37, 38, 39, 40, 41] PCs. PCs from these tissues express contractile proteins, can generate force, and have integrated calcium signaling across the vascular bed [31, 32, 42, 43]. However, the contractile properties of cardiac PCs have not been studied.

Furthermore, PCs have been shown to contribute to no‐reflow in *in vivo* rodent models [37, 38, 39]. No‐reflow is a concept that vessels no longer reperfuse an ischemic area even though the artery is reopened and there is no physical obstruction in the vessels [44, 45]. No‐reflow was initially thought to be caused by endothelial/myocardium inflammation and blockage of the vessel by leukocytes after ischemia until the discovery of the PCs' contribution [44, 45]. Most recently, it has been shown in an *in vivo* model where the areas of the blocked capillaries colocalized with PCs after a myocardial infarction (MI) [46]. In another *in vivo* study, Chintalgattu *et al*. found that sunitinib‐malate‐treated mice had fewer PCs, which led to a decrease in coronary perfusion and a decline in cardiac function. These *in vivo* studies thus led us to ask whether cardiac PCs can contract and regulate cardiac perfusion under normal physiological conditions. Additionally, we wanted to investigate how do cardiac PCs behave under *in vitro* pathophysiological conditions such as a hypoxic environment, elevated glucose levels to mimic a diabetic prone environment, and elevated low‐density lipoprotein (LDL) levels to mimic an atherosclerotic prone environment.

Here, we isolated PCs marked by NG2^+^ PDGFRβ^+^ CD146^+^ CD34^−^ CD31^−^ CD45^−^ from mouse hearts. The cardiac PCs contained the genes and proteins necessary for cellular contraction in addition to actin and myosin. We show that cardiac PCs contract and relax to phenylephrine (PE) and adenosine stimulation, respectively. Furthermore, blockade of adrenergic and purinergic receptors inhibited the effects of PE and adenosine. Under hypoxic conditions, PCs are sensitive to hypoxia but more resilient than expected. When PCs were treated with supraphysiologically levels of LDL, the cells decreased in proliferation and formed lipid droplets. Under hyperglycemic conditions, PCs became proinflammatory. Taken together, our study provides evidence that cardiac PCs are vasoactive and respond to pathological conditions by compromising barrier integrity which can lead to defects in cardiac perfusion and function.

## Materials and methods

### Animals and ethics approval

Wild‐type C57/BL6J male mice were purchased from Jackson Laboratory (Bar Harbor, ME, USA). All studies and procedures were performed in accordance with the Amgen IACUC approved protocol and with standard ethical guidelines as those detailed by the Committee on Publication Ethics.

### Pericyte isolation and cell culture

The isolation method for primary cardiac pericytes (PCs) follows our previously published method (Lee *et al*. [47]). Please see publication for exact details. Briefly, mice were sedated under isoflurane anesthesia, and the hearts were perfused through the descending aorta with heparinized Dulbecco's PBS. The hearts were then harvested, masticated into smaller pieces, and dissociated with a final concentration of 500 µg·mL^−1^ collagenase B enzyme solution for 75 min. Dissociated cells were stained with an antibody cocktail containing anti‐mouse NG2‐AF488, CD31‐APC, CD140b‐PE, CD146‐BV605, CD34‐BV421, and CD45‐PE‐Cy7 at 1 : 100 dilution each and cell viability dye at 1 : 1000 dilution in staining buffer. The PC population was obtained through fluorescence cell sorting. Purified cells were then cultured and subcultured to expand the number of cells.

### Smooth muscle and endothelial cell cultures

Coronary smooth muscle cells (SMC) or ECs from C57/BL6J mice were commercially purchased (Cell Biologics Inc., Chicago, IL, USA) and cultured according to manufacturer's protocol until passage 7 for usage in experiments.

### Proliferation curve

Pericytes were seeded at 8000 cells/mL into two 6‐well cell culture plate (Corning Inc., Corning, NY, USA) precoated with gelatin (Cell Biologics Inc.) in either Dulbecco's modified Eagle media (DMEM) with 4.5 g·L^−1^ glucose, l‐glutamine, and sodium pyruvate (Corning Inc.) supplemented with +5% FBS (Corning Inc.) +1% penicillin/streptomycin (P/S) (Corning Inc.) or endothelial growth medium 2 (EGM‐2; Cell Biologics, Inc., Chicago, IL, USA) supplemented with the ECs medium supplement kit (Catalog M1168, Cell Biologics Inc., Chicago, IL, USA). Cells were seeded at 10 000 cells per well into a 24‐well cell culture plate (Corning) precoated with gelatin (Cell Biologics Inc.) in cell culture media. After 6 h for cells to attach, media was changed to include LDL (Sigma) or d‐glucose (Sigma). Plates were placed into an Incucyte Zoom instrument (EssenBioscience Inc., Ann Arbor, MI, USA) integrated with the incucyte zoom 2018A software (EssenBioscience Inc., Ann Arbor, MI, USA), and growth was monitored for 5 days to construct proliferation curves.

### Western blot

Passage 7 cardiac PCs or mouse coronary SMCs (Cell Biologics, Inc.) were grown till confluent in a 6‐well cell culture plate. Cells were then treated under normal media conditions, hypoxic conditions (2% O_2_), LDL (Sigma, St. Louis, MO, USA), or d‐glucose (Sigma) for 24 or 48 h. After treatment, cells were washed with ice‐cold DPBS (Corning Inc., Corning, NY, USA) and each well was lysed with 40 µL 1xRIPA buffer (Cell Signaling Technologies, Danvers, MA, USA). Lysed cells were triturated and spun down at 4 °C for 15 min at 19 000 x***g*** in a centrifuge. Bicinchoninic acid (Pierce Biotechnology, Waltham, MA, USA) was done on the supernatant for protein quantification. Samples were boiled for 5 min at 95 °C with 4× Laemmli sample buffer (Bio‐Rad Laboratories, Hercules, CA, USA). Fifty microgram of total protein was loaded onto 4–15% gradient precasted gels (Bio‐Rad Laboratories). SDS/PAGE was ran at 125 mV for 1.5 h and transferred onto 0.22 µm poly(vinylidene difluoride) at 0.17 mAmps for 2 h at 4 °C. Blot was blocked with 5% milk + TBST for 1 h at room temperature then incubated with primary antibody overnight at 4 °C in blocking buffer. Blot was washed three times and incubated with secondary antibody for 1 h at room temperature. Blot was washed three more times and then incubated for 5 min with West Dura substrate solution (Pierce Biotechnology, Waltham, MA, USA). Blot was exposed, and image was capture via a Bio‐Rad ChemiDoc or Li‐cor Odyssey. Blot was then stripped with stripping buffer (Pierce Biotechnology) and reprobed with multiple antibodies. See Table 1 for antibodies and dilutions used and Table 2 for treatment compounds.

**Table 1:** Antibodies used in all experiments.

Antibody	Supplier	Catalog number	Dilution	Application
NG2‐FITC	Millipore	AB5320A4	1 : 100	FACS
CD31‐APC (clone MEC 13.3)	BD	551262	1 : 100	FACS
CD140b‐PE (clone APB5)	eBioscience	12‐1402‐81	1 : 100	FACS
CD146‐BV605 (clone ME‐9F1)	BD	740434	1 : 100	FACS
CD34‐BV421 (clone RAM 34)	BD	56268	1 : 100	FACS
CD45‐PE‐Cy7 (clone 30‐F11)	BD	552848	1 : 100	FACS
Near IR Live/Dead Stain	Life Technologies	L10119	1 : 1000	FACS
NG2	Millipore	AB5320	1 : 1000	WB
α‐SMA	Abcam	ab32575	1 : 1000	WB
PDGFRβ	Cell Signaling Technology	3169S	1 : 1000	WB
GAPDH	Cell Signaling Technology	5174S	1 : 10 000	WB
α‐actinin	Abcam	ab18061	1 : 1000	WB
caveolin‐1	Abcam	ab2910	1 : 1000	WB
caldesmon	Abcam	ab32330	1 : 1000	WB
vimentin	Abcam	ab92547	1 : 1000	WB
calponin	Abcam	ab46794	1 : 1000	WB
y‐SMA	Abcam	ab123034	1 : 1000	WB
SMMHC11	Abcam	ab53219	1 : 1000	WB
MLC2	Cell Signaling Technology	3672S	1 : 1000	WB
Vinculin	Invitrogen	700062	1 : 1000	WB
Calsequestrin	Cell Signaling Technology	2891	1 : 1000	WB
α1a‐adrenergic receptor	Abcam	ab3462	1 : 400	WB
α1b‐adrenergic receptor	Abcam	ab45871	1 : 500	WB
β‐actin	Sigma	A2228‐100uL	1 : 5000	WB
Perilipin‐2	Novus	NB110‐40877	1 : 1000	WB
P‐GSK‐3β	Cell Signaling Technology	9322S	1 : 1000	WB
T‐GSK‐3β	Cell Signaling Technology	9315S	1 : 1000	WB
P‐Akt	Cell Signaling Technology	4060S	1 : 1000	WB
T‐Akt	Cell Signaling Technology	4691S	1 : 1000	WB
HIF‐1α	Abcam	ab179483	1 : 100/1 : 1000	ICC/WB
Annexin V	eBioscience	17‐8007‐74	1 : 20	FCM
Propodium Iodide	eBioscience	00‐6990‐42	1 : 20	FCM
Anti‐mouse HRP	Cell Signaling Technology	7076S	1 : 10 000	WB
Anti‐rabbit HRP	Cell Signaling Technology	7074S	1 : 10 000	WB

**Table 2:** Treatment compounds used in experiments.

Treatment/Compound	Supplier	Catalog number	Lot No
PE hydrochloride	Sigma	P1250000	N/A
Endothelin‐1	Tocris	1160	N/A
Adenosine	Sigma	A9251‐25G	WXBC5186V
Phentolamine methanesulfonate salt, 98%	Frontier Scientific	300598	LG50Q45
CGS‐15943	Tocris	1699	4A/212461
Antimycin A	Sigma	A8674‐25MG	096M4022V
Sodium iodoacetate	Sigma	I2512‐25G	SLBS5337
LDL	Sigma	L7914‐5MG	SLCB0768
d‐glucose	Sigma	G8270‐100G	SLBV7620
Insulin	R&D Systems	1544‐IR	SUY0816091

### Immunocytochemistry

Passage 7 cardiac PCs were grown in a 96‐well plate (PerkinElmer Cell Carrier, Perkin Elmer, Waltham, MA, USA) until 90% confluent. Cells were washed with warm 1× DPBS and fixed with 4% paraformaldehyde (Sigma) for 30 min at room temperature. Cells were washed three times with 1× DPBS and permeablized with 0.1% Triton X‐100 (Sigma) for 10 min at room temperature. Cells were then blocked with SuperBlock (Thermo Fisher Scientific, Waltham, MA, USA) for 1 h at room temperature. After blocking, anti‐hypoxia‐inducible factor 1 alpha (HIF‐1α) antibody (Abcam, Cambridge, UK) diluted at 1 : 100 with Superblock was added and incubated at 4 °C overnight. Next day, cells were washed three times with wash buffer (0.1× SuperBlock + 1× DPBS). Secondary anti‐rabbit HRP (Cell Signaling Technologies, Danvers, MA, USA) diluted 1 : 1000 in Superblock was added and incubated for 2 h at room temperature in the dark. Secondary antibody was washed off three times with wash buffer. A 1 : 1000 dilution of 300 µm DAPI (Life Technologies, Carlsbad, CA, USA) stain was added on for 5 min at room temperature. Cells were washed three times with 1xDPBS and mounted with ProLong Diamond (Life Technologies) mounting media. Cells were imaged on an Opera Phenix confocal (PerkinElmer) at 40×.

### Electrical cell intuitive sensing (ECIS)

Cardiac PCs or SMCs at P7 were seeded onto 96‐well 20idf plates (Applied Biophysics Inc., Troy, NY, USA) that were pretreated with cysteine solution (Applied Biophysics Inc.) and coated with gelatin (CellBiologics, Inc., Chicago, IL, USA). Cells were plated at 25 000 cells per well at 200 µL per well. Cells were settled at room temperature for 30 min before the plate was transferred to the ECIS ZTheta 96W module (Applied Biophysics Inc.) inside a cell incubator kept at 37 °C and 5% CO_2_. Measurements were made in the single frequency mode at 8000 Hz for 24 h. At the 24 h of time point, plate was removed from module, half of the media (100 µL) aspirated off, and treatments or pretreatments (such as inhibitors) of 100 µL at 2× concentration were added in. Plate was subsequently moved back into the incubator and measurements resumed for another 24 h. Analysis was done using the ecis software (Applied Biophysics Inc.). Impedance was normalized to the time point right before treatment was added. Delta impedance was measured at peak contraction or relaxation from the time point right before treatment was added. See Table 2 for treatment compounds used.

### Transendothelial electrical resistance (TEER)

Passage 7 mouse coronary artery ECs (Cell Biologics, Inc.) were grown on Transwell filters of 0.4‐µm pores with 6.5 mm diameter (Corning Inc.) at 1 000 000 cells/mL in 200 µL per well of EGM‐2 (Cell Biologics, Inc.). PCs were grown on the bottom of the Transwell filter adaptor (Applied Biophysics Inc.) coated with gelatin (Applied Biophysics Inc.) at 500 000 cells/mL in 1 mL of DMEM + 5% FBS + 1% P/S. Inserts were placed into a Transwell filter adapter and then moved to the ECIS Ztheta 16W array module (Applied Biophysics Inc., Troy, NY, USA) inside a cell incubator at 37 °C and 5% CO_2_. TEER measured for 48 h. After 48 h, media was changed and TEER was measured for another 48 h. Analysis was done by the ECIS software. Data were normalized to the cell‐free chamber.

### Transwell permeability assay

Endothelial cells were grown on Transwell filters (Corning Inc.) at 500 000 cells/mL in 250 µL per well of EGM‐2. PCs were grown on 24‐well plates at 25 000 cells/mL in 500 µL per well of DMEM. Inserts were placed into the 24 wells with and without PCs, and ECs were grown for 48 h alone or together with PCs. After 48 h, a new 24‐well plate was prepared with 500 µL of media of DMEM in each well. Media was removed from the filter wells and replaced with 25 µg·mL^−1^ of FITC‐Dextran at 40 kDa (Sigma) diluted in 150 µL per well of EGM‐2. Cells were incubated for 30 min back in the incubator. After 30 min, 100 µL media from each bottom well was transferred into the wells of an opaque black 96‐well plate in duplicates. Relative fluorescence units (RFU) measurements of the media from the bottom wells were made by EnVision (PerkinElmer) with excitation/emission at 485 nm/535 nm.

### RNA sequencing

Passage 5 PCs were grown to confluence in normal media (DMEM with 4.5 g·L^−1^ glucose, l‐glutamine, and sodium pyruvate (Corning Inc.) supplemented with + 5% FBS (Corning Inc.) + 1% P/S (Corning Inc.), and passage five SMCs were grown to confluence following manufacturer's protocol (CellBiologics). Both cell types were harvested from a T75 cm flask. Cell mRNA was isolated using RNase kit (Qiagen, Hilden, Germany) following manufacturer's protocol. Isolated mRNA was quantified using a NanoDrop, and mRNA quality was checked using Agilent Technologies 2100 Bioanalyzer. Samples were frozen on dry ice and shipped to Quick Biology (Quick Biology Inc. Pasadena, CA, USA www.quickbiology.com) for analysis. The reads were first mapped to the latest UCSC transcript set using Bowtie2 version 2.1.0, and the gene expression level was estimated using RSEM v1.2.15. TMM (trimmed mean of *M*‐values) was used to normalize the gene expression.

### qRT–PCR

Passage 7 PCs were grown till confluent in a 6‐well cell culture plate. Cells were then treated under normal media conditions, hypoxic conditions (2% O_2_), LDL (Sigma), or d‐glucose (Sigma) in fresh media for 24 or 48 h. After treatment finished, cells were washed with ice‐cold DPBS (Corning Inc.). Cell mRNA was isolated using RNase kit (Qiagen, Hilden, Germany) following manufacturer's protocol. Isolated mRNA was quantified using a NanoDrop. TaqMan probes were purchased and used with a qRT–PCR master mix (Agilent Technologies, Santa Clara, CA, USA). Samples were run on a Quant7 Flex System (Thermo Fisher Scientific) using the deltaCT protocol. Data were analyzed using the quantstudio7 software (Thermo Fisher Scientific). See Table 3 for probes used and Table 2 for treatment compounds.

**Table 3:** TaqMan probes used in qRT–PCR.

Gene	TaqMan Probe	UniGene	Species	Supplier	Catalog number
ADFP	Mm00475794_m1	Mm.381	Mouse	Thermo Fisher Scientific	4331182
PDGFRb	Mm00435553_m1	Mm.4146	Mouse	Thermo Fisher Scientific	4331182
IL6	Mm00446190_m1	Mm.1019	Mouse	Thermo Fisher Scientific	4331182
CCL2	Mm00441242_m1	Mm.290320	Mouse	Thermo Fisher Scientific	4331182
TNF‐α	Mm00443258_m1	Mm.1293	Mouse	Thermo Fisher Scientific	4331182
GAPDH	Mm9999915_g1	Mm.304088	Mouse	Thermo Fisher Scientific	4331182

### FLIPR

Passage 7 cells were grown at 10 000 cells per well in a 384‐well plate for 16 h in 25 µL per well to be interrogated with the FLIPR calcium 6 evaluation kit (Molecular Devices, San Jose, CA, USA). Cells were then incubated at 37 °C, 5% CO_2_ with the calcium fluorescence dye for 2 h in the incubator with 2.5 mm final concentration of probenecid (Sigma). Measurement of calcium flux was immediately done postaddition of PE (Sigma) or DMSO (Sigma) treatment by the FLIPR Tetra instrument system (Molecular Devices, San Jose, CA, USA). Delta max‐min of calcium fluorescence signal was calculated and plotted versus the log of the molar concentration.

### ELISA

Cells were seeded at sub confluency in a 6‐well plate (Corning Inc.) and placed in normal culturing conditions, 2% O_2_ hypoxic conditions, treated with LDL or d‐glucose at various concentrations. After 24 or 48 h, the culture media was assayed in duplicates in an ELISA for platelet‐derived growth factor bb (PDGFbb), vascular endothelial growth factor‐A (VEGF‐A), IL‐6, or C‐C motif chemokine ligand 2 (CCL2) (R&D Systems, Minneapolis, MN, USA) following manufacturer's protocol. See Table 2 for treatment compounds.

### Apoptosis

Cells were grown under normal culture conditions and placed into a 2% O_2_ hypoxia chamber when subconfluent in a T75 cm flask. After 48 h in hypoxia, cells were rinsed in warm DPBS and lifted with cell dissociation buffer (Gibco, Waltham, MA, USA). Cells were then washed with wash buffer and stained with Annexin V and propidium iodide following manufacturer's protocol for the Annexin V apoptosis detection kit APC (eBioscience Inc., San Diego, CA, USA). Cells were analyzed on a LSR flow cytometer interfaced with FACS Diva software. Data were analyzed with flowjo software (FlowJo LLC, Ashland, OR, USA).

### Chemical ischemia (CI)

The chemical ischemic substance used was a combination of antimycin A and sodium iodoacetate listed in Table 2. The high dose of CI consisted of 500 µm sodium iodoacetate and 5 µm antimycin A. The low dose of CI consisted of 5 µm sodium iodoacetate (Sigma) and 50 nm antimycin A (Sigma). Treatment is added as described in ECIS section.

### Cell viability

Cells were grown under hypoxic culture conditions in 96‐well culture plates with 10% well volume of resazurin dye (R&D Systems, Minneapolis, MN, USA). Every 24 h, a plate was removed from the hypoxic chamber and measured at 570 nm with wavelength correction at 600 nm in a spectrophotometer (Molecular Devices, San Jose, CA, USA).

### Lipid droplet staining

Cells were grown in 24‐well glass plates in 500 µL media for 24 h. Next day, cell media was changed to include either control media, 50 µg·mL^−1^ LDL, or 250 µg·mL^−1^ LDL. Cells were treated for 24 h. Then, cells were stained with an Oil Red O kit (BioVision Inc., Milpitas, CA, USA) for neutral lipids following manufacturer's protocol. Images of cells were taken at 10 and 40× magnification on an EVOS bright‐field microscope.

### Statistics

All statistics were done using graphpad prism 7 software (GraphPad Software Inc., San Diego, CA, USA). Two‐way ANOVA was done followed by Bonferroni's *post hoc* test where applicable with significance at *P* ≤ 0.05. One‐way ANOVA was done followed by Turkey's multiple comparison *post hoc* test where applicable with significance at *P* ≤ 0.05. Student's *t*‐test was done where significance was *P* ≤ 0.05.

## Results

### Isolation and phenotypic characterization of cardiac pericytes

**Fig. 1:**
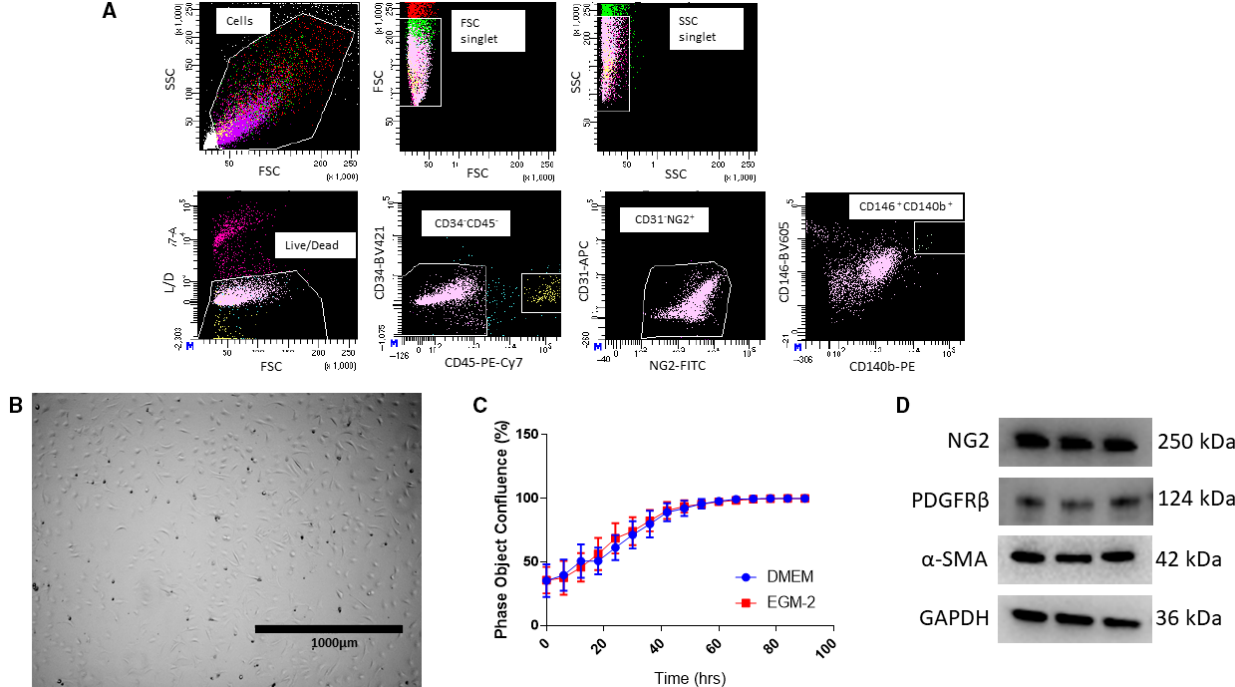
(A) Cells were isolated from murine hearts, stained with antibodies, and put through fluorescence‐activated cell sorting (FACS) and gated with negative markers for CD31/34/45 and positive markers for NG2, PDGFRβ, and CD146. (B) Bright‐field images of cultured cells passaged after isolation showing typical PC morphology. (C) Proliferation curve of primary PCs in DMEM and EGM‐2. There was no significant difference between the two medias used on their proliferation. (D) Protein expression of typical PC markers. Data are presented as the mean ± SD. Scale bar = 1000 µm.

Cardiac PCs are not available commercially, and they need to be isolated from a primary source. Utilizing our previously published protocol [47], we isolated a population of PCs that are NG2^+^ PDGFRβ^+^ CD146^+^ CD34^−^ CD31^−^ CD45^−^ from mouse heart (Fig. 1A). In addition to the expression of canonical PC markers, the cells remained a homogenous population with minimal observable change in morphology throughout the passages (Fig. 1B). In the literature, others have used different types of media to culture their primary PCs. As shown in Fig. 1C, there was no significant difference in growth rate when the cells were cultured with DMEM + 5% FBS + 1% P/S or EGM‐2 + 5% FBS + 1% P/S. Lastly, our cells express typical PC markers such as NG2, PDGFRβ, and αSMA (Fig. 1D). These results indicate that cardiac PCs can be prospectively isolated and maintained *in vitro* for detailed disease modeling.

### Functional characterization of cardiac pericytes

**Fig. 2:**
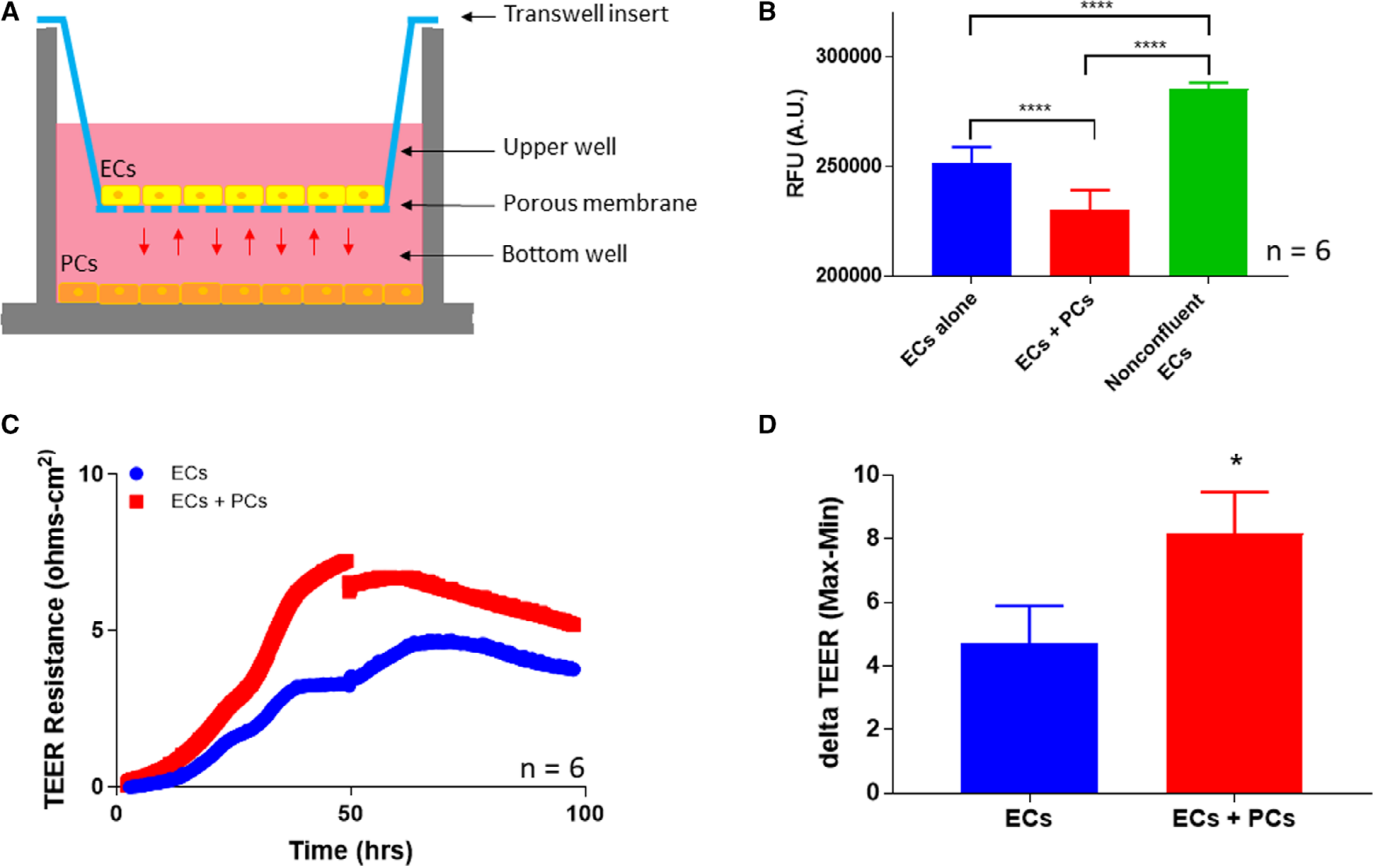
(A) Cartoon representation of experimental set‐up where mouse coronary ECs were cocultured with cardiac PCs through a Transwell filter for 24 h. (B) 25 µg·mL^−1^ of FITC‐Dextran was added to the top well for 30 min. RFU were measured in the bottom well. There was a 1.29‐fold significant decrease in permeability in the coculture system (*n* = 6, **** *P* ≤ 0.0001, one‐way ANOVA). (C) Graphical representation of when PCs and ECs were cocultured and TEER was measured. (D) After 48 h, TEER increased significantly by 2.3‐fold in the cocultured wells compared to the monocultured wells (*n* = 6, * *P* ≤ 0.01, Student's *t*‐test). Data are presented as the mean ± SD.

To determine whether the isolated population were PCs with the ability to increase barrier integrity, we utilized transwell assays to assess the effects of the PCs on ECs (Fig. 2A). Here, we used mouse coronary ECs cocultured with the cardiac PCs. ECs were cultured in the upper well while PCs were cultured in the bottom well. In this barrier integrity assay, FITC bound 40 kDa dextran is placed in the upper well for 30 min, and then, fluorescence of the bottom well is measured. The fluorescence found in the bottom well represents how leaky the endothelial monolayer is. Coculturing with PCs decreased the permeability of the 40 kDa FITC‐Dextran significantly by 1.3‐fold (*P* = 0.0001; Fig. 2B). As a control, in a nonconfluent layer of EC, the permeability increased significantly when comparing with the confluent layer with the nonconfluent layer (*P* = 0.0001; Fig. 2B). In our second assay to assess barrier integrity, we used the same transwell set‐up, but we measured the transendothelial electrical resistance, also known as TEER, of the endothelial monolayer over time (Fig. 2C). An increase in resistance is an indication of increased barrier integrity. Electrodes were placed in the wells, and cell behavior is recorded in real time. Consistent with the dextran diffusion assay, there is a significant 2.3‐fold decrease in TEER when comparing the coculture to the monoculture after 48 h (*P* = 0.0383; Fig. 2D). Therefore, PCs cocultured with ECs were able to increase EC barrier integrity which indicate that cardiac PCs share similar functions to typical PCs.

### Cardiac pericyte transcriptome and molecular structure

**Fig. 3:**
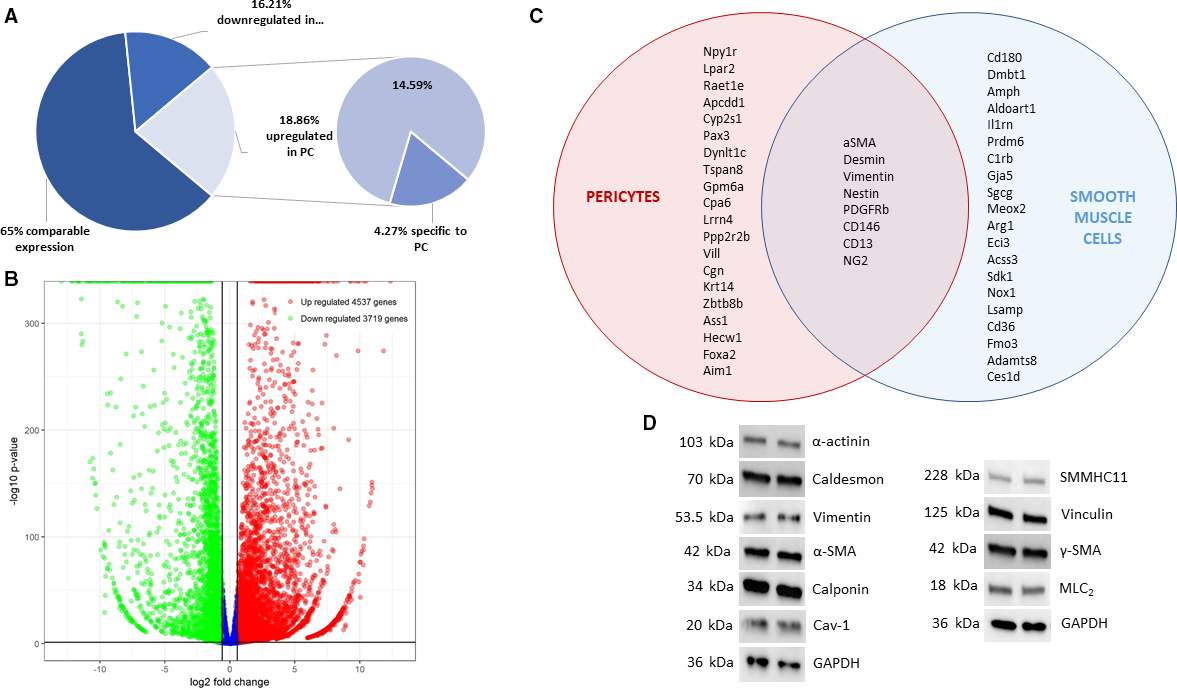
Molecular characterization of primary cardiac PCs. (A) Percentage of significant differentially expressed genes of cardiac PCs when compared with SMC in RNA‐seq (*n* = 3). (B) Volcano plot depicting the spread of significantly upregulated and downregulated genes of cardiac PCs when compared with SMC. (C) Venn diagram depicting genes expressed solely by each cell type from RNA‐seq and the current accepted markers for PCs which are also expressed by SMC. (D) Protein expression of contractile proteins.

To further characterize and validate our primary isolated cardiac PCs are not SMC, RNA‐seq was performed on cardiac PCs and commercially purchased mouse coronary SMCs followed by differential gene expression analysis. Of the total sequences read, about 35% of the sequences were differentially expressed where 19% were upregulated in PCs and 16% were downregulated in PCs. Furthermore, about 4% of the upregulated genes were PC specific (Fig. 3A). The volcano plot depicts the spread of the genes that were significantly upregulated and downregulated in cardiac PCs in comparison with SMCs (Fig. 3B). The Venn diagram depicts several of the genes that were solely specific to each cell type with currently known markers that are in expressed in both cell types (Fig. 3C). These data suggest that our population of PCs have a unique transcriptome profile that is different from SMCs.

The microenvironment of microvessels could contain vasoactive factors that cause cardiac PC contraction [36]. However, cardiac PCs have been not shown to contract *in vitro,* and we wanted to investigate if cardiac PCs even can do so. From the RNA‐seq results of our cardiac PCs, Table 4 shows that these cells express genes of the thin, thick, and intermediate filaments and the contractile apparatus. Furthermore, the cells also express the proteins that are required for contraction and relaxation (Fig. 3D). These data suggest that cardiac PCs express some of the essential proteins necessary for contraction and relaxation.

**Table 4:** Contractile proteins separated by categories of their gene expression from RNA‐seq. The sequence reads have been log‐transformed.

Category	Gene	PC	SMC
Thin filaments	actin, alpha 2, smooth muscle, aorta	2228.126377	970.9103762
	actin, gamma 2, smooth muscle, enteric	60.15007041	3.651576677
Thick filaments	myosin, heavy polypeptide 11, smooth muscle	0.536776257	8.407627578
	myosin, light chain 12A, regulatory, nonsarcomeric	493.8085537	818.958498
	myosin, light chain 12B, regulatory	67.22209386	314.2666743
Intermediate filaments	vimentin	1834.020965	3332.303797
	desmin	0.621214693	0.358305314
	vinculin	1205.75204	495.8974362
	actinin, alpha 1	660.122412	705.9825083
	actin, beta	3361.47022	6279.302377
Contractile apparatus	tropomyosin 1, alpha	2319.112132	411.2959054
	tropomyosin 2, beta	245.9005131	891.1982213
	tropomyosin 3, gamma	519.8432779	261.4541382
	tropomyosin 4	1331.063047	1266.608366
	calponin 1	44.358496	1.544161439
	calponin 2	407.1057595	284.7032821
	calponin 3, acidic	637.7093129	651.2683946
	caldesmon 1	2599.716194	2530.047688
	myosin light chain kinase 3	2.25236037	1.0475104
	myosin light chain kinase family, member 4	0.21190905	2.699624555

### Phenylephrine‐induced calcium flux

**Fig. 4:**
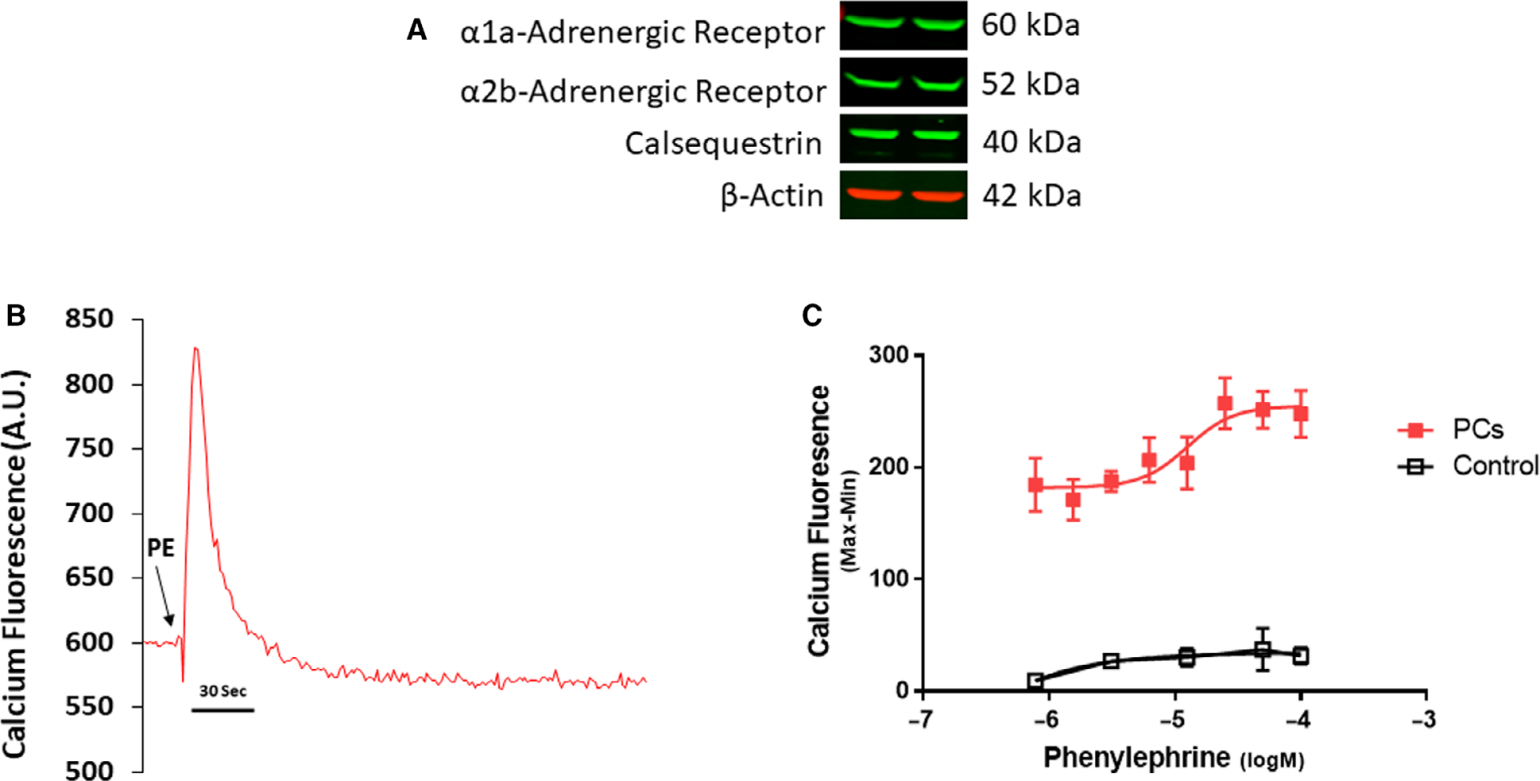
Calcium flux upon α‐adrenergic pathway activation in cardiac PCs. (A) Protein expression of contractile components of the α‐adrenergic pathway. FLIPR assay was used to determine calcium flux. (B) Representative image of calcium flux in response to PE. (C) Dose response of cells to PE. Control is the cells treated with DMSO at the same dilution as PE (*n* = 8). Data are presented as the mean ± SD.

From RNA‐seq, the gene expression for α‐adrenergic receptors was high compared to other conical pathways for contraction (data not shown). We found that the cells expressed proteins of the α‐adrenergic pathway (Fig. 4A). An indication of α‐adrenergic agonist activation of the receptor is calcium flux. The cells are dose‐dependently sensitive to PE as indicated by their calcium response curve (Fig. 4B,C). The α‐adrenergic receptor is active and could potentially mediate PC contraction.

### Contraction and relaxation to phenylephrine and adenosine

**Fig. 5:**
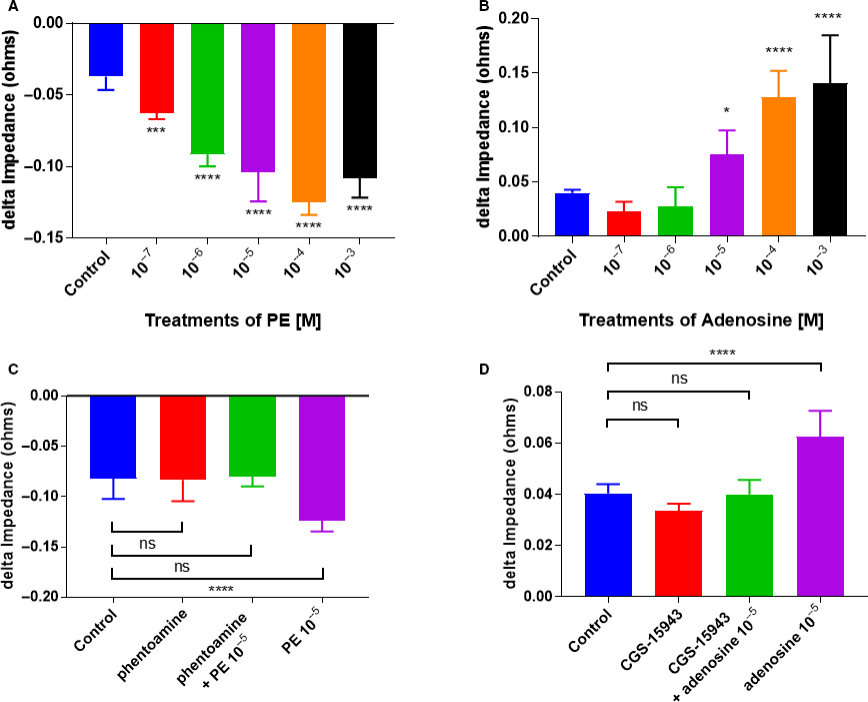
ECIS measurements of real‐time cell behavior. Quantitation of the delta max‐min of impedance to a dose response of cells to (A) PE (*n* = 6, *** *P* = 0.0008; **** *P* ≤ 0.0001; *P* ≤ 0.0001; *P* ≤ 0.0001; *P* ≤ 0.0001, one‐way ANOVA) and (B) adenosine (*n* = 6, *P* = 0.4751; *P* = 0.7496; * *P* = 0.0211; **** *P* ≤ 0.0001; *P* ≤ 0.0001, one‐way ANOVA). Quantitation of the delta max‐min of impedance to a dose response of cells to (C) α‐adrenergic blocker (*n* = 6, *P* = 0.9957, *P* = 0.9796, **** *P* ≤ 0.0001, one‐way ANOVA) and (D) adenosine blockade by CGS‐15943 (*n* = 6, *P* = 0.1370, *P* = 0.9997, **** *P* ≤ 0.0001, one‐way ANOVA). Data are presented as the mean ± SD.

The ECIS platform measures cell behavior in real time [48, 49]. Here, treating PCs with increasing doses of PE showed a dose‐dependent decrease in cell impedance. As the PCs contracted upon PE treatment, there was a significant decrease in cell impedance due to less cell surface contact with the electrodes (*P* = 0.0008; *P* ≤ 0.0001; *P* ≤ 0.0001; *P* ≤ 0.0001; *P* ≤ 0.0001; Fig. 5A). Conversely, PCs treated with the relaxant adenosine, there was more cell surface contact with the electrodes resulting in dose‐dependent increases in cell impedance (*P* = 0.4751; *P* = 0.7496; *P* = 0.0211; *P* ≤ 0.0001; *P* ≤ 0.0001; Fig. 5B).

To further validate whether the observed changes in impedance were specifically due to agonist stimulation, we used receptor antagonists to counteract contraction and relaxation. We preincubated the cells with an α‐adrenergic blocker, phentolamine methane, for 1 h before PE 10^−5^ m treatment with no significant changes in impedance (*P* = 0.9957). We observed a substantial decrease in impedance upon PE treatment like Fig. 5A (*P* ≤ 0.0001) while the cells pretreated with phentolamine methane did not have a significant change in impedance (*P* = 0.9796; Fig. 5C).

Similarly, we preincubated the cells with an adenosine receptor blocker, CGS‐15943, for 1 h before adenosine 10^−5^ m treatment. There was no significant effect of the pretreatment when compared to the control (*P* = 0.1370). Similar to Fig. 5, we saw a significant increase in impedance upon adenosine treatment (*P* ≤ 0.0001) while the cells treated with CGS‐15943 did not show any significant changes in impedance (*P* = 0.9997; Fig. 5D). In short, inhibition of the receptors by receptor antagonists abolished PC response to agonist stimulation. These data indicate that cardiac PCs respond accurately to external stimuli for contraction and relaxation.

### Response to hypoxia

**Fig. 6:**
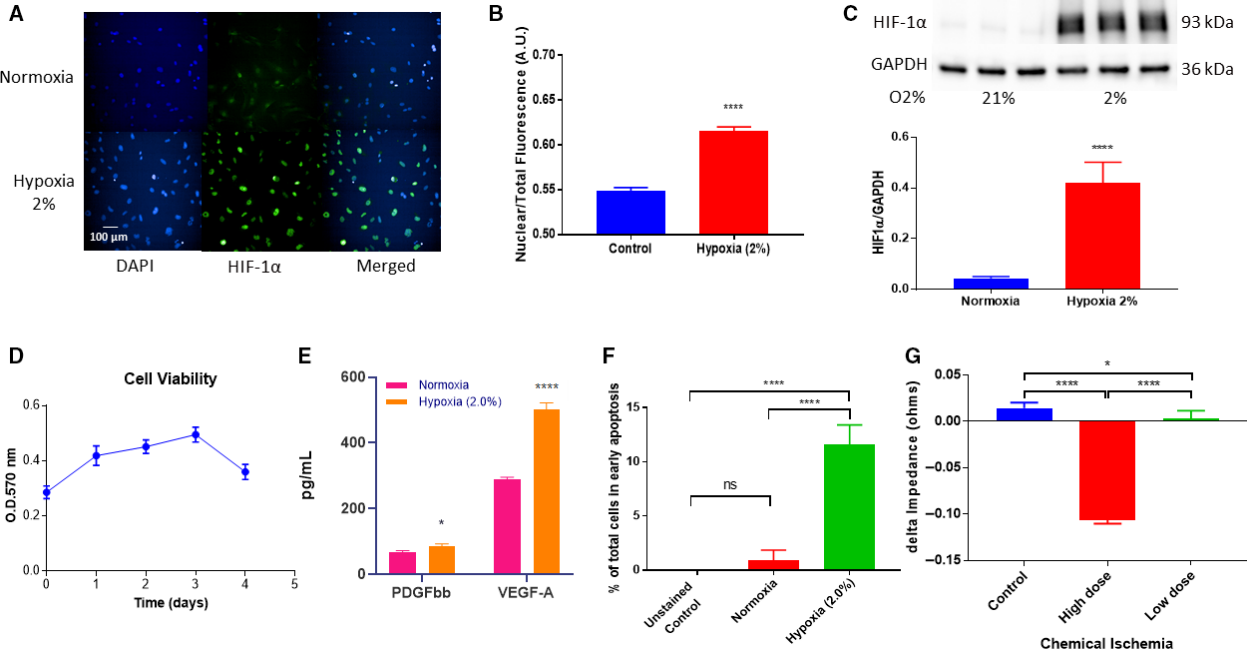
Cardiac PCs were subjected to hypoxic conditions (2%) for 24 or 48 h. (A) HIF‐1α translocation after 24 h (B) Quantification of nuclear to total fluorescence of HIF‐1α protein (*n* = 4, **** *P* ≤ 0.0001, Student's unpaired *t*‐test). (C) HIF‐1α protein expression increased significantly after 24 h normalized to GAPDH (*n* = 6, **** *P* ≤ 0.0001, Student's unpaired *t*‐test). (D) Cell viability was determined over the course of 4 days. Angiogenic factors (E) PDGFbb (*n* = 8, * *P* = 0.0005 Student's unpaired *t*‐test) and VEGF‐A (*n* = 8, **** *P* ≤ 0.0001, Student's unpaired *t*‐test) were significantly increased after 48 h. (F) Apoptosis was determined by AV5 expression and PI staining where there was a significant difference between normoxic and hypoxic conditions after 48 h (*n* = 3, *P* = 0.7802, **** *P* ≤ 0.0001, *P* ≤ 0.0001, one‐way ANOVA). ECIS measurements of real‐time cell behavior. Quantitation of the delta max‐min of impedance to a dose response of cells to (G) chemical ischemia (*n* = 6, * *P* = 0.0144, **** *P* ≤ 0.0001, *P* ≤ 0.0001, one‐way ANOVA). Data are presented as the mean ± SD. Scale bar = 100 µm.

In no‐reflow, a hypoxic environment created from a MI could lead to PC death [38, 46, 50, 51]. Subjecting the PCs to 2% hypoxia activated the master hypoxic transcription regulator, HIF‐1α. After 24 h in hypoxia, we observed a translocation of the inactive HIF‐1α protein from the cytosol to the nucleus (Fig. 6A). Quantification of the cytosolic‐to‐nuclear signal ratio was significant in hypoxia when compared with normoxia (*P* ≤ 0.0001; Fig. 6B). Protein expression of HIF‐1α also significantly increased under hypoxic conditions after 24 h (*P* = 0.0001; Fig. 6C). We then asked, how long can these cells survive under hypoxia? Evaluation of their viability using resazurin showed that they start to die between the 3‐ and 4‐day marks. (Fig. 6D). What can be adding to their survival? We detected a significant increase in secretion of PDGFbb and VEGF‐A by PCs (*P* = 0.0115 and *P* ≤ 0.0001, respectively) under hypoxic conditions (Fig. 6E). VEGF‐A and PDGFbb are well‐established factors in hypoxia‐induced angiogenesis that are regulated by HIF‐1α. After 48 h in hypoxia, we detected a significant difference in the number of cells entering the early stages of apoptosis when compared with cells cultured in normoxic conditions (*P* ≤ 0.0001; Fig. 6F). Therefore, under hypoxic conditions, the HIF‐1α signaling pathway is potentially controlling pathways for PC cell survival, angiogenesis, and apoptosis.

### Hypoxia‐induced cellular contraction

We saw eventual cell death upon prolonged hypoxia treatment. Our next question was whether hypoxia caused or prevented contraction. We used two different doses of our chemical ischemia treatment, which we labeled as high and low. Our chemical ischemia treatment consists of a combination of antimycin A and sodium iodoacetate which depletes the cells of energy by inhibiting the mitochondrial light chain. After 1.5 h of the chemical ischemia treatment, the low dose significantly decreased cell impedance (*P* = 0.0144) and the high dose significantly further decreased cell impedance (*P* ≤ 0.0001) when compared with control (Fig. 6G). The difference between the low and high dose was also significant (*P* ≤ 0.0001; Fig. 6G). These results indicate that PCs contract in response to hypoxia conditions.

### LDL treatment and lipid droplet formation

**Fig. 7:**
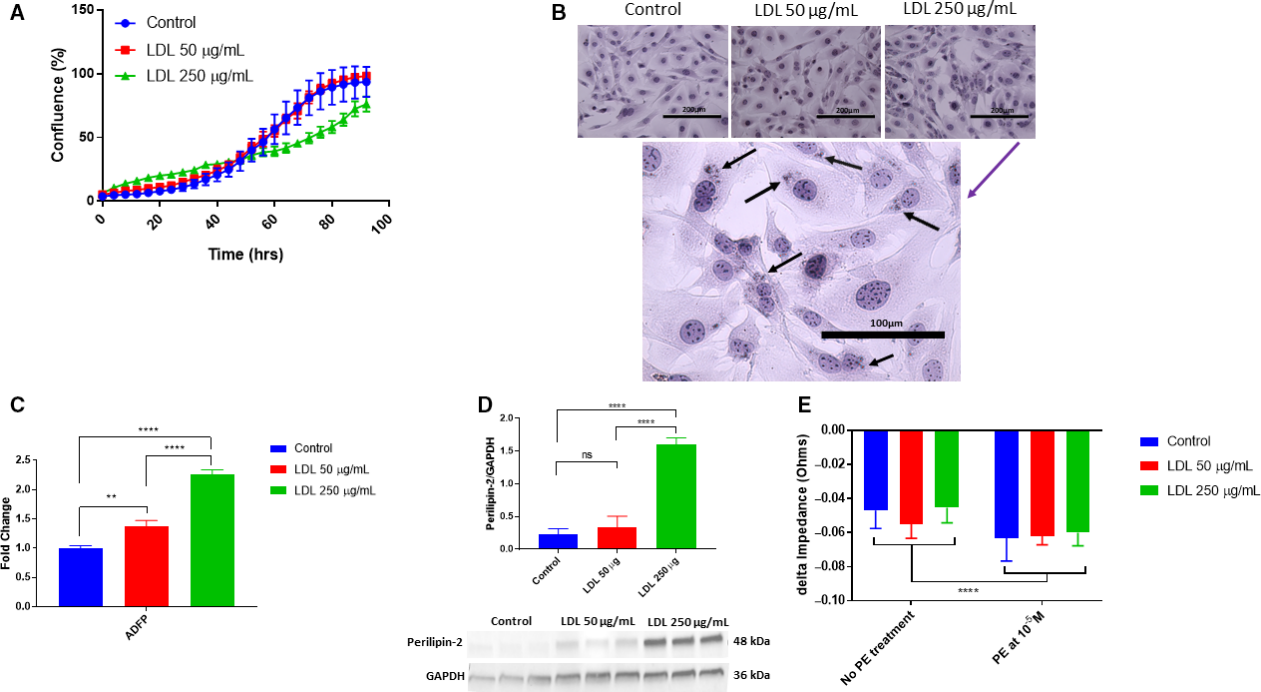
Effects of 24 h of LDL treatment on cardiac PCs. (A) Cell proliferation decreased upon high LDL treatment at 250 µg·mL^−1^ but no changes at 50 µg·mL^−1^. (B) Cardiac PCs formed lipid droplets (neutral lipid staining with Oil Red O and counterstained with hematoxcylin) in response to 24 h of LDL treatment. Images taken at 10×. Black arrows point to lipid droplets in the cytoplasm at 40x after treatment at 250 µg·mL^−1^. (C) Fold change in mRNA levels normalized to GAPDH. ADFP mRNA levels increased significantly upon increasing concentrations of LDL treatment (*n* = 3, ** *P* = 0.0012, **** *P* < 0.0001, *P* < 0.0001, one‐way ANOVA). (D) Protein expression normalized to GAPDH. There is a significant increase in perilipin‐2 protein expression upon LDL treatment at 250 µg·mL^−1^ but not at 50 µg·mL^−1^ (*n* = 3, *P* = 0.5173, **** *P* < 0.0001, *P* < 0.0001, one‐way ANOVA). ECIS measurements of real‐time cell behavior. (E) Quantitation of the delta max‐min of cell impedance to LDL treatment at 50 or 250 µg·mL^−1^ with or without PE‐induced contraction (*n* = 6, **** *P* < 0.0001, two‐way ANOVA). Data are presented as the mean ± SD. Scale bar = 200 µm for 10× images. Scale bar = 100 µm for 40× image.

In an atherosclerotic environment, cells typically experience lipotoxicity [52]. Because of the close interaction between PCs and ECs, we hypothesized that PCs will react to high levels of lipids, in particular, LDL. Under supraphysiological LDL levels of 250 µg·mL^−1^, rate of PC proliferation was diminished (Fig. 7A). When PCs were stained with Oil Red O (neutral lipids), we found that high levels of LDL caused the cells to form lipid droplets when compared with control or low levels of LDL at 50 µg·mL^−1^ (Fig. 7B). Further confirming lipid droplet accumulation, adipose differentiation factor protein (ADFP) mRNA was significantly upregulated (*P* = 0.0012 and *P* ≤ 0.0001, respectively), concomitant with a dose–response increase when comparing the 50 and 250 µg·mL^−1^ doses (*P* ≤ 0.0001). Moreover, perilipin‐2 protein level was also significantly increased at 250 µg·mL^−1^ when compared to control (*P* ≤ 0.0001) but not at 50 µg·mL^−1^ when compared to control (*P* = 0.5173) (Fig. 7C,D). In addition to being a marker for lipid droplets, ADFP upregulation could potentially be an indicator of adipogenesis as suggested in the name of the protein. Elevated LDL levels mimicking hypercholesterolemia potentially induce a morphology change (lipid droplet formation) in PCs.

### LDL levels do not affect pericyte contraction

If PCs were undergoing adipogenesis, their ability to contract may be impaired. Using the ECIS platform, we found that there was a significant difference in cell impedance after LDL treatment of the cells at 50 or 250 µg·mL^−1^ when compared with control (*P* ≤ 0.0001; Fig. 7E) However, we found that the LDL treatments, enough to induce an increase expression in ADFP mRNA and protein levels, did not hinder the cell's ability to contract upon PE stimulation when compared with control (*P* = 0.5094). Lastly, the interaction between LDL treatments with and without PE stimulation was not significant (*P* = 0.8256). Therefore, PC retained their ability to contract upon PE stimulation even under conditions of elevated LDL.

### High glucose treatment prevents cellular contraction

**Fig. 8:**
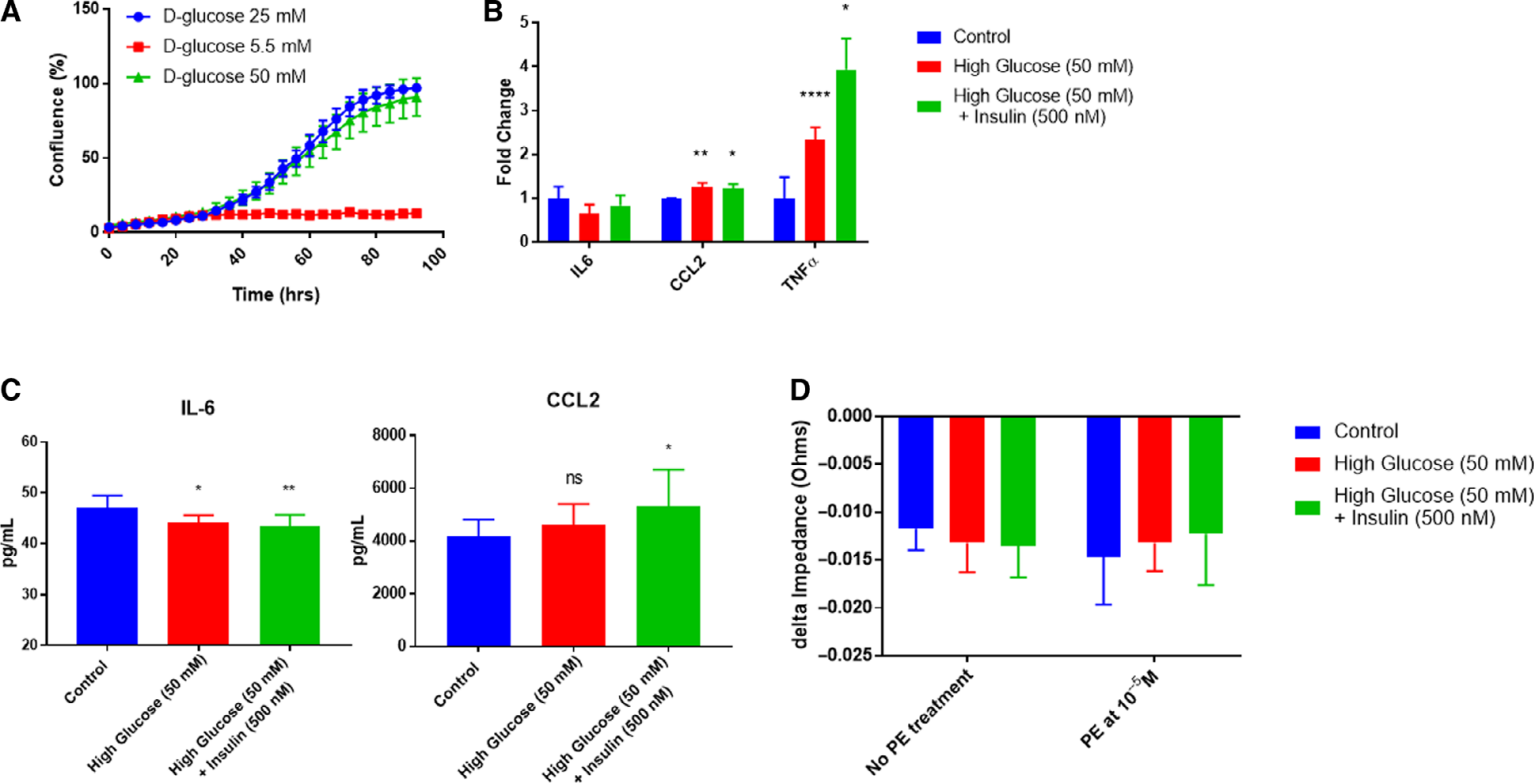
Effects of 24 h of elevated d‐glucose levels on cardiac PCs. (A) Cell proliferation was unaffected by increased glucose levels at 50 mm but cells died at low glucose levels of 5.5 mm. (B) Fold change in mRNA levels of common cytokines in diabetic conditions normalized to GAPDH. IL‐6 did not have any changes upon high glucose or high glucose in combination with insulin treatment (*n* = 3, *P* = 0.1016, one‐way ANOVA). CCL2 increased significantly upon high glucose treatment without and with insulin (*n* = 3, ** *P* = 0.01154 and * *P* = 0.02269, one‐way ANOVA). TNF‐α increased significantly upon high glucose treatment without and with insulin (*n* = 3, **** *P* = 0.00751 and * *P* = 0.000738, one‐way ANOVA). (C) Protein expression of common cytokines. IL‐6 protein levels decreased significantly upon high glucose treatment with and without insulin (*n* = 3, ** *P* = 0.0064 and * *P* = 0.0359, one‐way ANOVA). CCL2 protein levels did not significantly increase upon high glucose treatment but it did significantly increase upon high glucose treatment with insulin (*n* = 3, *P* = 0.2457 and * *P* = 0.0472, one‐way ANOVA). ECIS measurements of real‐time cell behavior. (D) Quantitation of the delta max‐min of cell impedance to high glucose or high glucose plus insulin treatment with or without PE‐induced contraction (*n* = 14, *P* = 0.4841, *P* = 0.9230, *P* = 0.0901, two‐way ANOVA). Data are presented as the mean ± SD.

Lastly, we characterized the cells under high glucose treatment to be in a more a diabetic prone environment. Decreasing the amount of glucose in the media significantly altered the cell's ability to survive and proliferate. However, increasing the amount of glucose did not change the proliferation rate (Fig. 8A). We first looked at mRNA levels of common cytokines in diabetes [53] under high levels of glucose with and without insulin. We found no significant change in IL‐6 mRNA levels upon glucose stimulation nor upon glucose and insulin treatment when compared with control (*P* = 0.1016; Fig. 8B). However, CCL2 and tumor necrosis factor alpha (TNF‐α) mRNA levels were significantly increased upon glucose stimulation with insulin (*P* = 0.02269 and *P* = 0.000738, respectively) and without insulin treatment (*P* = 0.01154 and *P* = 0.00751, respectively) when compared with control (Fig. 8B). At the protein level, IL‐6 had a significant decrease upon glucose stimulation both with insulin and without insulin (*P* = 0.0064 and *P* = 0.0359, respectively) when compared with control (Fig. 8C). TNF‐α protein levels were undetectable. However, CCL2 had a significant increase in protein levels upon glucose stimulation with insulin (*P* = 0.0472) but was not substantial without insulin (*P* = 0.2457) when compared with control (Fig. 8C). PCs had an increase in inflammatory cytokines upon treatment of high glucose levels.

We had investigated what happens to PC contraction upon high doses of chemical ischemia and LDL, and lastly, we wanted to examine what happens to PC contraction under high glucose levels. High glucose levels with and without insulin did not cause a significant increase in PE‐induced PC contraction (*P* = 0.5024 and *P* = 0.9134, respectively) nor their interaction (*P* = 0.0901; Fig. 8D). The PC ability to contract upon PE stimulation was diminished after treatment of elevated glucose levels.

## Discussion

Prior to our work, cardiac PCs have been shown to contribute to angiogenesis, vascular integrity, and perfusion [1, 5, 13, 14]. Moreover, the loss of PC in the heart leads to compromised vascular integrity, hypertrophy, and decreased cardiac function [19]. However, the PCs' contribution to cardiovascular homeostasis and their contractile properties remains to be determined. Here, we isolated cardiac PCs and for the first time, characterized their contractile properties and showed their response to different *in vitro* model conditions that play a part in human disease conditions. Our *in vitro* data suggest that cardiac PCs possess the ability to contract and relax under normal physiological conditions to vasoactive molecules and different disease conditions alter their ability to contract. Therefore, cardiac PCs can modulate microvascular tone, perfusion, and pressure.

Our isolated population of cardiac PCs are characterized by NG2^+^ PDGFRβ^+^ CD146^+^ CD34^−^ CD31^−^ CD45^−^ (Fig. 1). Our published isolation methodology has been optimized to isolate these primary murine cardiac PCs [47]. We recognize that these cells are cultured and passaged and can possibly have a change in phenotype; however, after passaging the cells to P7, there were no observable phenotypic changes (marker expression and morphology) as previously published [47]. In the literature, many studies on PCs from other organs only use canonical markers such as NG2^+^ [4, 54, 55] and PDGFRβ^+^ [20, 54, 56, 57] for identification. We used a culmination of positive (NG2, PDGFRβ, and CD146) and negative markers (CD31, CD34, and CD45) found in the literature [1, 3, 11, 28]. Unlike PCs found in skeletal muscle [58, 59], it is currently unknown how many subtypes of PCs, if any, are in the heart. Further characterization and identification of cardiac PCs will need to be done to investigate the homogeny of PCs in the heart [60]. Moreover, our primary cardiac PCs behave functionally in coculture with ECs like PCs found in other tissues by influencing endothelial integrity through paracrine signaling (Fig. 2) [13, 60, 61, 62]. Our stringent marker panel in combination with our functional experiments provides evidence that these are indeed cardiac PCs.

Thus far, we have not been able to find data on the transcriptome of cardiac PCs. Here, we have performed RNA‐seq to show the differential gene expression profile from SMCs to further validate that our PCs are not SMCs (Figs 3 and S1). Similar to publications comparing the transcriptome profile of brain PCs and vascular SMCs [63, 64, 65], our isolated cardiac PC population has a unique transcriptome profile from SMCs. Our RNA‐seq differential gene expression analysis results revealed that genes with the highest fold change such as Npy1r, Lpar2, Raet1e, Apcdd1, and Cyp2s1 were solely expressed by cardiac PCs and not by SMCs (Fig. 3). These genes encode proteins that are G‐coupled receptors, glycoproteins, and an enzyme. The unique expression of one or more the proteins that are encoded by these genes can potentially be used as a novel marker for mouse cardiac PCs.

Until recently, regulation of blood flow and pressure is only known to be controlled by SMCs [41, 66, 67, 68, 69, 70]. It is well established that neural‐humoral vasoactive factors modulate the contraction and relaxation of SMCs such as nitric oxide, adrenergic/purinergic receptor agonists. However, the localization of PCs puts them at sympathetic terminals where they can also receive these neurohumoral signals [71]. Our transcriptome data revealed that cardiac PCs have a high number of adrenergic receptors, and unlike brain and retinal PCs, they do not express endothelin receptors. ET‐1 signaling in cortical PCs has been shown to induce calcium flux and cellular constriction [31]. PE, which is an α‐adrenergic receptor agonist, similarly activated the signal transduction pathway as indicated by a dose‐dependent response in calcium flux (Fig. 4).

The quantification of cell behavior based on cell impedance has been used to study cardiomyocyte contraction, and its' use has been extended to study PC contraction [35]. We find that cardiac PCs contract and relax *in vitro* upon stimulation with PE and adenosine (Figs 5 and S4–S5). We have also provided human brain PC and mouse SMC data as positive controls and mouse EC data as a negative control (Figs S2 and S3). This technique assumes that changes in impedance reflect changes in cellular contraction and relaxation due to changes in cell surface contact with the electrodes [35]. The delta impedance we observed with the relaxant adenosine is similar to brain PC literature [35]. The difference in kinetics between the cardiac and brain PCs could be due to the expression levels of the α‐adrenergic receptors on each cell type or that they are derived from different organs altogether. Our data provide evidence that cardiac PCs are vasoactive and could possibly regulate capillary tone and pressure.

Under hypoxic conditions, such as during a MI, PCs are one of the first cells to experience the deprivation of nutrients and oxygen due to their anatomical location [38, 50, 71]. Pathways and mechanisms that modulate the PCs' response to hypoxia are currently unknown but as speculated by us and others, HIF‐1α can modulate cell survival and apoptosis (Fig. 6) [72, 73, 74, 75]. PCs have been shown to be resistant to hypoxic insult *in vitro* [61, 76, 77, 78]. Like brain PCs [61, 73], Fig. 6D suggests that cardiac PCs are pretty resilient to hypoxia and this could be due to the PC's mesenchymal origin [3, 65, 79, 80] or the increased secretion of HIF‐1α mediated [73, 74] cell survival factors that we and others have observed [61, 76, 77, 78]. This *in vitro* observation agrees with the *in vivo* study where cardiac PCs did not die in a 45min ischemic attack [46]. Rather, we believe that a longer ischemic attack will be needed to cause a majority of PC death to alter hemodynamics. HIF‐1α could also be mediating the apoptosis pathway [75, 81, 82] where PCs in culture have entered the early stages of apoptosis (Fig. 6F). Evidently, during chemical ischemia treatment, the decrease in impedance never returned to baseline (Fig. 6G). This could be due to the cells being in a permanent contracted state or cell death.

Next, we investigated the cardiac PC's response to an atherosclerotic environment (elevated LDL levels). Studies have suggested that perivascular cells like the PC can be adipogenic progenitors [83, 84]. We found that our cardiac PCs formed cytoplasmic lipid droplets with an increase in ADFP (marker for adipogenic potential) expression at both the mRNA and protein levels (Fig. 7). It has been previously shown in retinal PCs that they form lipid droplets and can be converted into immature adipocytes [85]. However, lipid droplets are also a site of lipid storage which could alter lipid metabolism due to lipotoxicity and play a part in the inflammatory response in atherosclerosis. Also, notably, lipid treatment did not hinder PE‐stimulated PC contraction (Fig. 7). Since our cells retained their pericytic properties, it is possible that cardiac PCs can contribute to lesion formation in the atherosclerotic process but are not adipogenic.

Our cardiac PCs show signs of inflammation after 24 h in high glucose media (Fig. 8). Moreover, PE‐stimulated contraction was blunted under the elevated glucose conditions. Retinal PCs and their contractile properties have been highly studied [29, 30] and characterized in diabetic neuropathy. Retinal PCs have been shown to be inhibited by high glucose media *in vitro* [86, 87]. Elevated glucose levels have been shown in retinal PCs to alter membrane voltage regulation through NKA which can potentially explain why there was no significant change in impedance when our cardiac PCs were stimulated with PE (Fig. 8) [88]. Furthermore, retinal PCs' responsiveness to endothelin‐1 was significantly decreased by elevated glucose levels which can affect the cells' contraction. Although our cardiac PCs do not express endothelin‐1 receptors, elevated glucose levels can also cause the cardiac PCs to develop PE resistance [89]. Therefore, the inability of cardiac PCs to contract under hyperglycemic conditions can contribute to the altered hemodynamics and hypertrophy seen in diabetics.

## Conclusions

In summary, our study shows for the first time in an *in vitro* model that primary cardiac PCs do contract and relax to vasoactive compounds. Their sensitivity to hypoxia and response to sympathetic stimulus can both contribute to the no‐reflow phenomena observed after a MI. Under high LDL levels, they may contribute to an atherosclerotic prone environment, and under elevated glucose levels, they may contribute to the cardiovascular disease risk in diabetes. Therefore, PCs can play an integral role in cardiac hemodynamics by regulating blood flow and pressure at the microvessel and capillary level in both normal physiological and pathophysiological states.

## Conflict of interest

The authors declare no conflict of interest.

## Author contributions

LLL, AYK, and VC conceived and designed the project. LLL acquired and analyzed the data. LLL, AYK, and VC interpreted the data. LLL wrote the manuscript. AYK and VC edited the manuscript.

## Supporting information


**Fig. S1.** RNA‐seq differential gene analysis. (a) Gene ontology enrichment (b) KEGG pathway enrichment.
**Fig. S2.** ECIS measurements of real‐time cell behavior of human brain pericytes and mouse coronary endothelial cells. (a) Plot of human brain pericyte impedance versus time to phenylephrine contraction, adenosine relaxation, and endothelin‐1 contraction. (b) Quantitation of the delta max‐min of impedance to a dose response of cells to phenylephrine contraction, adenosine relaxation, and endothelin‐1 contraction (*n* = 6, *P* = 0.0199, *P* = 0.0011, *P* ≤ 0.0001, One‐way ANOVA). (c) Plot of mouse coronary endothelial cell impedance versus time to phenylephrine contraction, adenosine relaxation, and endothelin‐1 contraction. (d) Quantitation of the delta max‐min of impedance to a dose response of cells to phenylephrine contraction, adenosine relaxation, and endothelin‐1 contraction (*n* = 6, *P* ≤ 0.0001, *P* = 0.9954, *P* = 0.1671, One‐way ANOVA). Data are presented as the mean ± SD.
**Fig. S3.** ECIS measurements of real‐time cell behavior. Plot of mouse coronary smooth muscle cells impedance versus time to (a) phenylephrine contraction and (c) adenosine relaxation. Quantitation of the delta max‐min of impedance to a dose response of cells to (b) PE (*n* = 6, *P* = 0.1059, *P* = 0.0378, *P* < 0.0001, *P* < 0.0001, *P* < 0.0001, One‐way ANOVA) and (d) adenosine (*n* = 6, *P* = 0.9031, *P* = 0.9619, *P* = 0.1855, *P* = 0.0004, *P* = 0.0004, One‐way ANOVA). Data are presented as the mean ± SD.
**Fig. S4.** ECIS plot of cardiac pericytes impedance versus time to (a) phenylephrine contraction, (b) adenosine relaxation, (c) α‐adrenergic blocker, and (d) adenosine blockage.
**Fig. S5.** ECIS plot of cardiac pericytes impedance versus time to (a) chemical ischemia (b) LDL treatment (c) glucose treatment with and without insulin.

## Data Availability

The RNA‐seq dataset is publicly available through the figshare repository using the following https://doi.org/10.6084/m9.figshare.12185118.
