# Supplementary figures and images for: Cardiac pericytes function as key vasoactive cells to regulate homeostasis and disease
Source: FEBS Open Bio. 2020 Dec 14;11(1):207–25. doi: 10.1002/2211-5463.13021 (PMC7780101; doi:10.1002/2211-5463.13021)

S1a

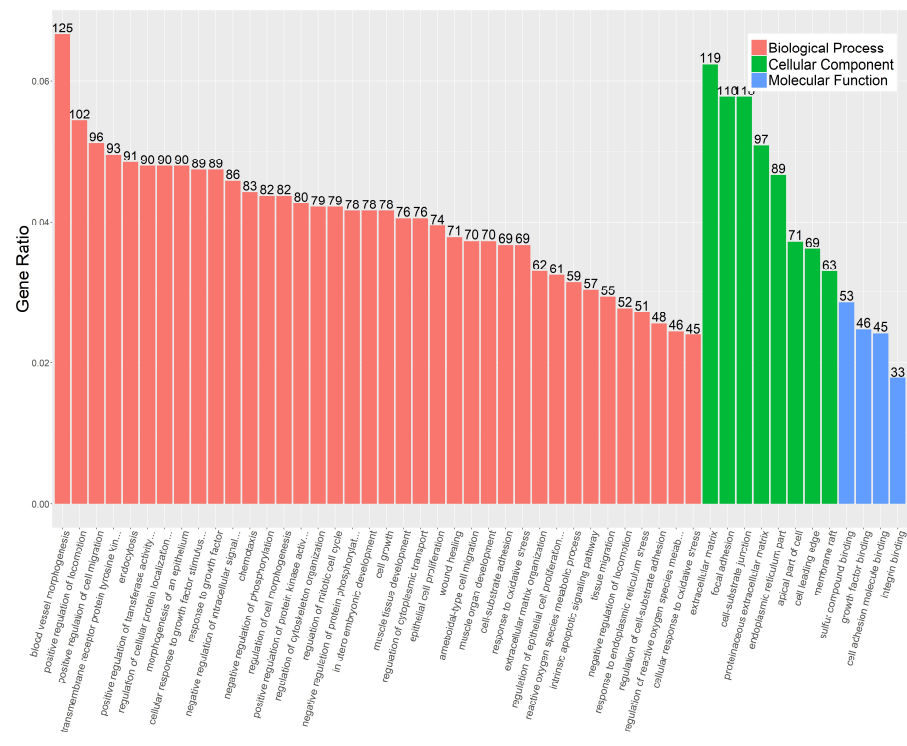

S1b

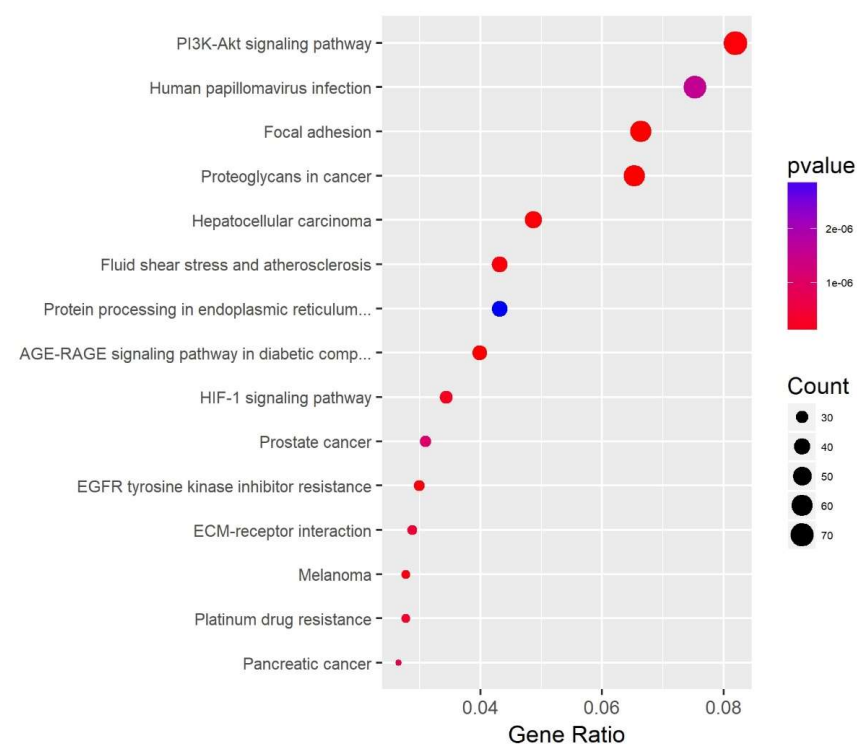

S2a

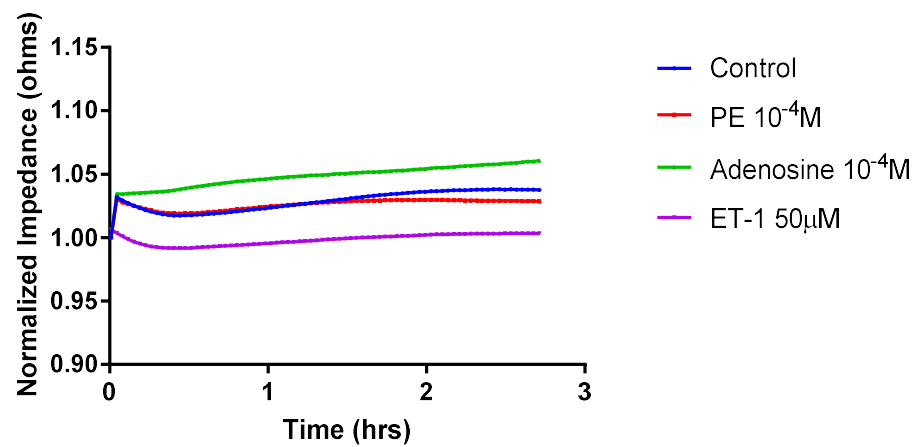

S2b

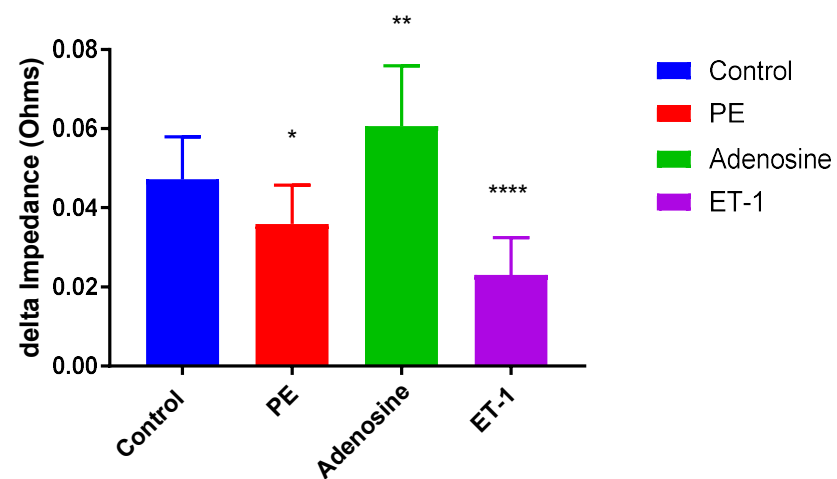

S2c

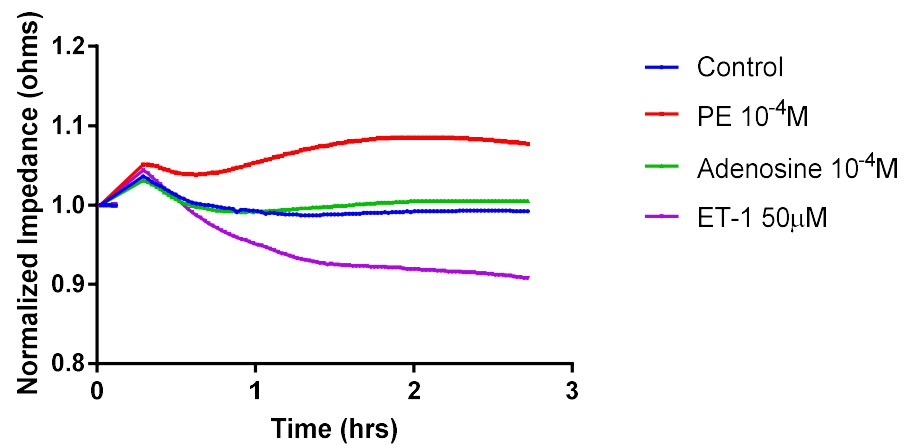

S2d

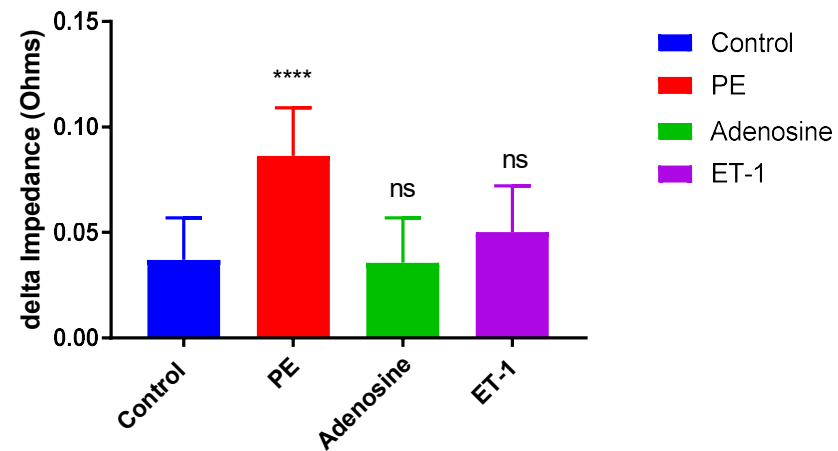

S3a

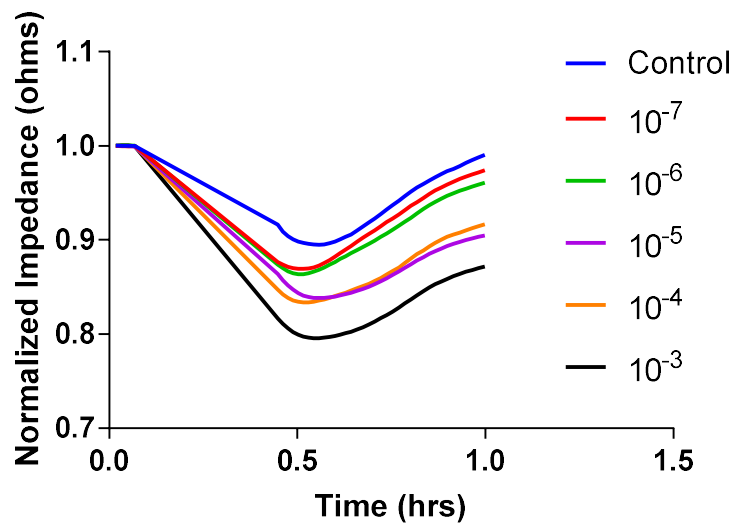

S3b

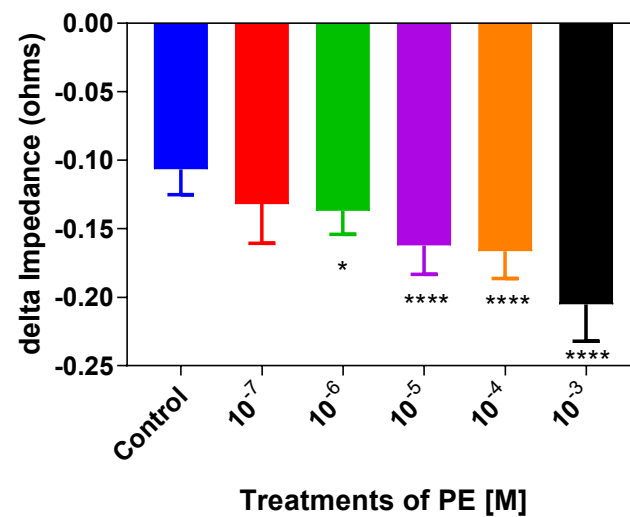

S3c

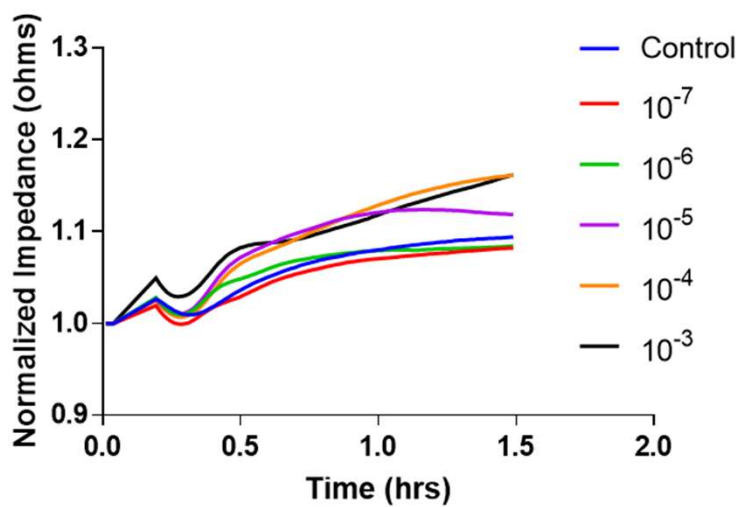

S3d

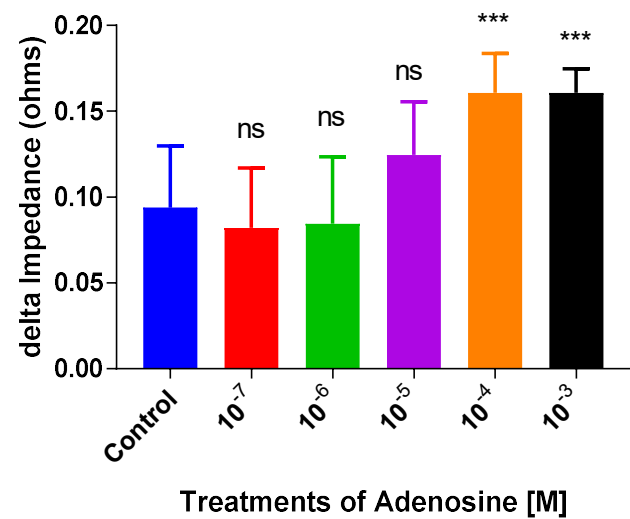

S4a

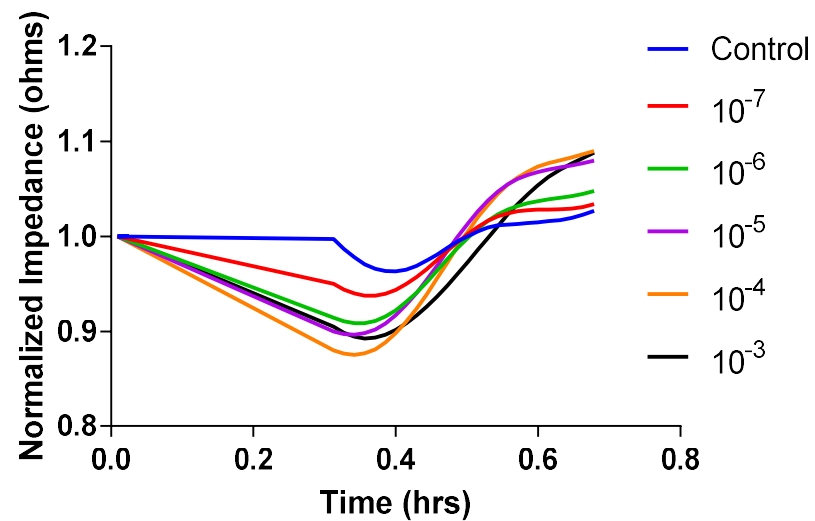

S4b

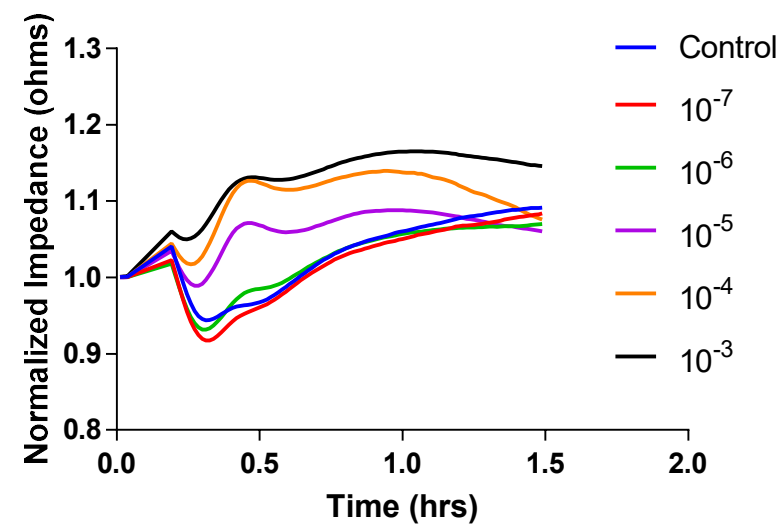

S4c

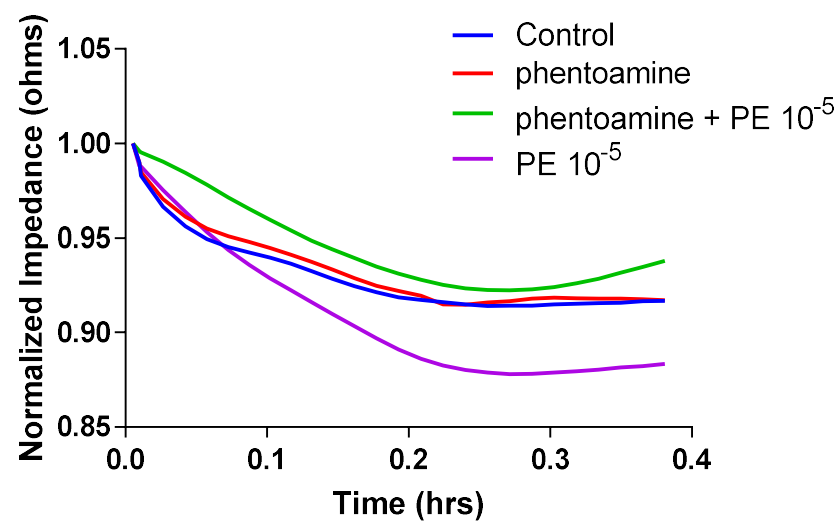

S4d

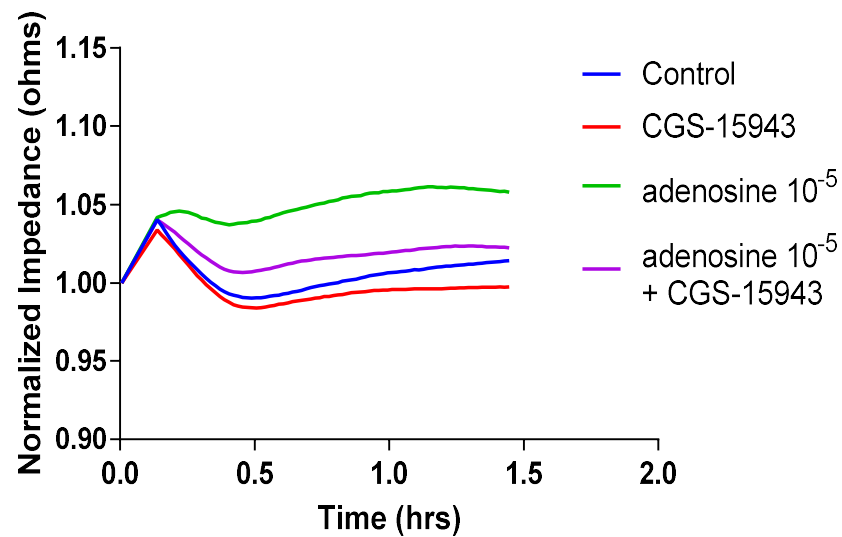

S5a

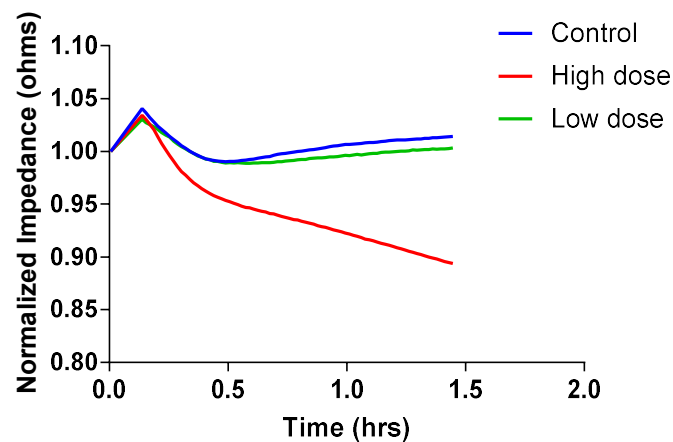

S5b

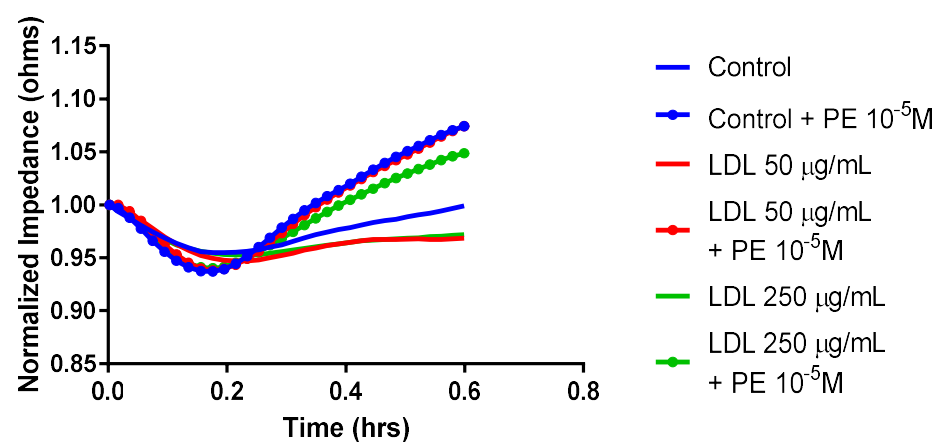

S5c

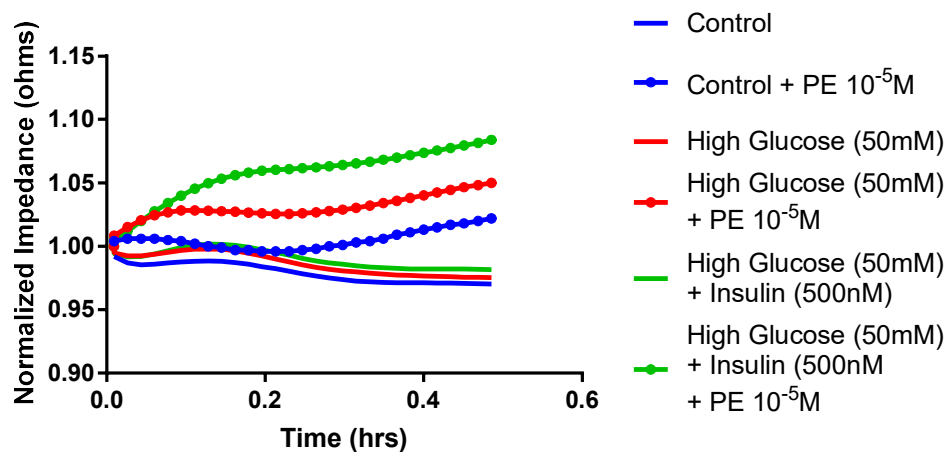

Supplement: Supplementary file 1 — Fig. S1. RNA‐seq differential gene analysis. (a) Gene ontology enrichment (b) KEGG pathway enrichment. Fig. S2. ECIS measurements of real‐time cell behavior of human brain pericytes and mouse coronary endothelial cells. (a) Plot of human brain pericyte impedance versus time to phenylephrine contraction, adenosine relaxation, and endothelin‐1 contraction. (b) Quantitation of the delta max‐min of impedance to a dose response of cells to phenylephrine contraction, adenosine relaxation, and endothelin‐1 contraction (n = 6, P = 0.0199, P = 0.0011, P ≤ 0.0001, One‐way ANOVA). (c) Plot of mouse coronary endothelial cell impedance versus time to phenylephrine contraction, adenosine relaxation, and endothelin‐1 contraction. (d) Quantitation of the delta max‐min of impedance to a dose response of cells to phenylephrine contraction, adenosine relaxation, and endothelin‐1 contraction (n = 6, P ≤ 0.0001, P = 0.9954, P = 0.1671, One‐way ANOVA). Data are presented as the mean ± SD. Fig. S3. ECIS measurements of real‐time cell behavior. Plot of mouse coronary smooth muscle cells impedance versus time to (a) phenylephrine contraction and (c) adenosine relaxation. Quantitation of the delta max‐min of impedance to a dose response of cells to (b) PE (n = 6, P = 0.1059, P = 0.0378, P < 0.0001, P < 0.0001, P < 0.0001, One‐way ANOVA) and (d) adenosine (n = 6, P = 0.9031, P = 0.9619, P = 0.1855, P = 0.0004, P = 0.0004, One‐way ANOVA). Data are presented as the mean ± SD. Fig. S4. ECIS plot of cardiac pericytes impedance versus time to (a) phenylephrine contraction, (b) adenosine relaxation, (c) α‐adrenergic blocker, and (d) adenosine blockage. Fig. S5. ECIS plot of cardiac pericytes impedance versus time to (a) chemical ischemia (b) LDL treatment (c) glucose treatment with and without insulin. [file FEB4-11-207-s001.pdf]
